# Supplementary material for: Pyruvate Dehydrogenase Kinase 1 inhibition mediated oxidative phosphorylation enhancement in cartilage promotes osteoarthritis progression
Source: BMC Musculoskelet Disord. 2023 Jul 20;24:597. doi: 10.1186/s12891-023-06585-6 (PMC10357736; doi:10.1186/s12891-023-06585-6)
Supplement: Supplementary file 1 — Additional file 1. [file 12891_2023_6585_MOESM1_ESM.zip › BP.Gsea.1653623667859/GOBP_POSITIVE_REGULATION_OF_SIGNAL_TRANSDUCTION_BY_P53_CLASS_MEDIATOR.html]

Details for gene set GOBP\_POSITIVE\_REGULATION\_OF\_SIGNAL\_TRANSDUCTION\_BY\_P53\_CLASS\_MEDIATOR[GSEA]

|  || Dataset | 55457\_collapsed\_to\_symbols.cls.cls #OA\_versus\_normal.cls.cls #OA\_versus\_normal\_repos |
| Phenotype | cls.cls#OA\_versus\_normal\_repos |
| Upregulated in class | normal |
| GeneSet | GOBP\_POSITIVE\_REGULATION\_OF\_SIGNAL\_TRANSDUCTION\_BY\_P53\_CLASS\_MEDIATOR |
| Enrichment Score (ES) | -0.61303514 |
| Normalized Enrichment Score (NES) | -1.9840859 |
| Nominal p-value | 0.0 |
| FDR q-value | 0.035732288 |
| FWER p-Value | 0.338 |
Table: GSEA Results Summary

  

Fig 1: Enrichment plot: GOBP\_POSITIVE\_REGULATION\_OF\_SIGNAL\_TRANSDUCTION\_BY\_P53\_CLASS\_MEDIATOR      
 Profile of the Running ES Score & Positions of GeneSet Members on the Rank Ordered List

  

| SYMBOL | TITLE | RANK IN GENE LIST | RANK METRIC SCORE | RUNNING ES | CORE ENRICHMENT || 1 | ING4 | inhibitor of growth family member 4 [Source:HGNC Symbol;Acc:HGNC:19423] | 680 | 0.598 | 0.0122 | No |
| 2 | SPRED2 | sprouty related EVH1 domain containing 2 [Source:HGNC Symbol;Acc:HGNC:17722] | 5179 | 0.058 | -0.3308 | No |
| 3 | ATM | ATM serine/threonine kinase [Source:HGNC Symbol;Acc:HGNC:795] | 6179 | -0.019 | -0.4063 | No |
| 4 | EEF1E1 | eukaryotic translation elongation factor 1 epsilon 1 [Source:HGNC Symbol;Acc:HGNC:3212] | 6296 | -0.027 | -0.4124 | No |
| 5 | ANKRD1 | ankyrin repeat domain 1 [Source:HGNC Symbol;Acc:HGNC:15819] | 6416 | -0.037 | -0.4177 | No |
| 6 | PML | PML nuclear body scaffold [Source:HGNC Symbol;Acc:HGNC:9113] | 6421 | -0.037 | -0.4140 | No |
| 7 | CDK5RAP3 | CDK5 regulatory subunit associated protein 3 [Source:HGNC Symbol;Acc:HGNC:18673] | 6583 | -0.048 | -0.4212 | No |
| 8 | PLA2R1 | phospholipase A2 receptor 1 [Source:HGNC Symbol;Acc:HGNC:9042] | 7321 | -0.103 | -0.4672 | No |
| 9 | HEXIM1 | HEXIM P-TEFb complex subunit 1 [Source:HGNC Symbol;Acc:HGNC:24953] | 8293 | -0.178 | -0.5233 | No |
| 10 | UBB | ubiquitin B [Source:HGNC Symbol;Acc:HGNC:12463] | 9142 | -0.243 | -0.5627 | No |
| 11 | RPL37 | ribosomal protein L37 [Source:HGNC Symbol;Acc:HGNC:10347] | 9265 | -0.253 | -0.5447 | No |
| 12 | PMAIP1 | phorbol-12-myristate-13-acetate-induced protein 1 [Source:HGNC Symbol;Acc:HGNC:9108] | 10084 | -0.329 | -0.5724 | Yes |
| 13 | RPS7 | ribosomal protein S7 [Source:HGNC Symbol;Acc:HGNC:10440] | 10608 | -0.384 | -0.5713 | Yes |
| 14 | PYHIN1 | pyrin and HIN domain family member 1 [Source:HGNC Symbol;Acc:HGNC:28894] | 10635 | -0.386 | -0.5313 | Yes |
| 15 | EIF5A | eukaryotic translation initiation factor 5A [Source:HGNC Symbol;Acc:HGNC:3300] | 11127 | -0.446 | -0.5209 | Yes |
| 16 | ATR | ATR serine/threonine kinase [Source:HGNC Symbol;Acc:HGNC:882] | 11231 | -0.461 | -0.4788 | Yes |
| 17 | RPS20 | ribosomal protein S20 [Source:HGNC Symbol;Acc:HGNC:10405] | 11507 | -0.502 | -0.4456 | Yes |
| 18 | RPS15 | ribosomal protein S15 [Source:HGNC Symbol;Acc:HGNC:10388] | 11671 | -0.527 | -0.4010 | Yes |
| 19 | CHD5 | chromodomain helicase DNA binding protein 5 [Source:HGNC Symbol;Acc:HGNC:16816] | 11776 | -0.545 | -0.3498 | Yes |
| 20 | MSX1 | msh homeobox 1 [Source:HGNC Symbol;Acc:HGNC:7391] | 12054 | -0.609 | -0.3051 | Yes |
| 21 | HIC1 | HIC ZBTB transcriptional repressor 1 [Source:HGNC Symbol;Acc:HGNC:4909] | 12118 | -0.624 | -0.2421 | Yes |
| 22 | RPL23 | ribosomal protein L23 [Source:HGNC Symbol;Acc:HGNC:10316] | 12558 | -0.777 | -0.1918 | Yes |
| 23 | MYC | "MYC proto-oncogene, bHLH transcription factor [Source:HGNC Symbol;Acc:HGNC:7553]" | 12801 | -0.970 | -0.1052 | Yes |
| 24 | DDX5 | DEAD-box helicase 5 [Source:HGNC Symbol;Acc:HGNC:2746] | 12834 | -1.038 | 0.0051 | Yes |
Table: GSEA details [plain text format]

  

Fig 2: GOBP\_POSITIVE\_REGULATION\_OF\_SIGNAL\_TRANSDUCTION\_BY\_P53\_CLASS\_MEDIATOR      
 Blue-Pink O' Gram in the Space of the Analyzed GeneSet

  

Fig 3: GOBP\_POSITIVE\_REGULATION\_OF\_SIGNAL\_TRANSDUCTION\_BY\_P53\_CLASS\_MEDIATOR: Random ES distribution      
 Gene set null distribution of ES for **GOBP\_POSITIVE\_REGULATION\_OF\_SIGNAL\_TRANSDUCTION\_BY\_P53\_CLASS\_MEDIATOR**
